# Rural Latino parent and child physical activity patterns: family environment matters

**DOI:** 10.1186/s12889-021-12085-w

**Published:** 2021-11-08

**Authors:** Benjamin Domogalla, Linda K. Ko, Reo Jones, Wafaa Bin Ali, Edgar Rodriguez, Catherine Duggan, Cynthia K. Perry

**Affiliations:** 1 School of Nursing, Oregon Health & Science University, 3455 SW US Veterans Hospital Rd, Portland, OR 97239 USA; 2 Division of Public Health Sciences, Fred Hutchinson Cancer Research Center, Seattle, WA USA; 3 Department of Health Services, University of Washington, Seattle, WA USA; 4 College of Nursing-Jeddah, King Saud Bin Abdulaziz University for Health Science, Jeddah, Saudi Arabia; 5 Therapeutic Products Program, Fred Hutchinson Cancer Research Center, Seattle, WA USA; 6 Department of Public Health, Fred Hutchinson Cancer Research Center, Seattle, WA USA

**Keywords:** Physical activity, Sedentary, Rural, Latino/Hispanic, Family

## Abstract

**Background:**

Rural Latino children and adults are less active than urban and non-Latino counterparts. We examined physical activity (PA) patterns of rural Latino children and their parents, and explored parental beliefs about and reported barriers of Latino family physical activity. Latino families in a rural area in eastern Washington state, with children in grades 3–5 were included.

**Methods:**

We used mixed methods. Children (*n* = 27) and parents (*n* = 25) wore an accelerometer for 5 days; parents (*n* = 31) participated in a semi-structured interview and completed a demographic survey. Parent and child activity levels were compared using paired t-tests; interviews were analyzed with qualitative content analysis.

**Results:**

Although 100% children and 46% parents met physical activity guidelines, parents and children spent most of the day in sedentary behaviors. Parent-reported PA barriers included their long work hours, lack of transportation, and their child’s screen-time.

**Conclusion:**

Addressing barriers and reducing sedentary time could increase PA of rural Latino families.

**Supplementary Information:**

The online version contains supplementary material available at 10.1186/s12889-021-12085-w.

## Background

Engaging in regular physical activity (PA) can reduce the incidence of chronic illness and certain cancers [1]. United States guidelines recommend adults engage in at least 150 min of moderate-intensity PA, or 75 min of vigorous-intensity PA each week and children and adolescents engage in moderate-vigorous PA (MVPA) for at least 60 min daily [2]. Most Americans do not meet recommended levels [3].

Latino children and adults are less physically active than other ethnic groups [4–7]. Children living in urban areas are approximately three times more active than rural children [8]. In 2017, urban adults met the combined aerobic and muscle-strengthening physical activity guidelines 25.3% of the time. However, rural adults only met the same guidelines 19.4% of the time [9]. Additionally, rural adults are proportionately more inactive than urban adults (62.8% versus 59.3%) [10]. These disparities in PA have been attributed to environmental policy, land use, culture, and socioeconomic status across the United States [11, 12].

Parental efforts to engage in PA with their children, parental attitudes towards being physically active, and their ability to access resources to maintain active lifestyles are key factors in the development of children’s PA [4, 13, 14]. For example, among urban Latino parent-preschool aged children dyads, the parents’ level of PA and sedentary behaviors were highly correlated with their children’s PA and sedentary behaviors [15]. Little is known about rural Latino parent-child PA patterns as well as how rural Latino families integrate PA recommendations into their daily lives.

Understanding PA patterns and parental influences on rural Latino families can inform the development of interventions designed to promote PA among this underserved and high-risk population. Here, we explore PA patterns of rural Latino parents and school-aged children, and parental beliefs about and reported barriers of Latino family PA, using a mixed-methods design.

## Methods

### Design

A parallel mixed methods study design in which quantitative and qualitative data were collected simultaneously [16], was conducted between August and December 2014.

### Setting and sample

This study was part of a larger community-based participatory research project aimed at addressing childhood obesity, conducted in four predominantly Latino (74–90% of population) rural agricultural towns in Washington state [17] with a median household income from $29,135 -$39,850 and 22–37% families living below the poverty level [18]. The Rural-Urban Commuting Area codes ranged from 4.2 to 7.0 [17]. Rural Latino families with a child enrolled in grades 3–5 in one of local elementary schools were eligible. Bicultural/bilingual (Spanish/English) community health workers (CHW) recruited parent-child dyads through community and school events and word of mouth.

The Fred Hutchinson Cancer Research Center Institutional Review Board approved this study. All participants signed an informed assent or consent form. Data were collected between August and December 2014. Families were given $150 for their participation in the study, including data collection not reported here.

### Quantitative data

#### Physical activity

PA was measured using wGT3x-BT Actigraph accelerometers (Pensacola, FL, USA). Parents and children were instructed to wear the accelerometer for the same 7 days consecutively for at least 10 h per day. Accelerometer data was captured in 30 s epochs. Actilife software (6.11.5, ActiGraph, Pensacola, FL, USA) was used to categorize activity counts into sedentary, light, moderate and vigorous activity using age-based thresholds [19, 20].

#### Demographic questionnaire

Parents completed a baseline questionnaire asking about their age, sex, employment, income, county of birth, language spoken, number of children in household and child’s age, and sex.

#### Height and weight

Parent and child height and weight were measured in triplicate using a calibrated stadiometer and scale respectively in the home, prior to the semi-structured interview. Body mass index (BMI) was calculated from height and weight for parents and CDC growth charts by age and gender were used to determine child’s BMI percentile.

#### Data analysis

All analyses were completed in SAS, 9.4. We calculated the average hours per day, overall and by day of the week children and parents spent in sedentary, light, moderate and vigorous PA. Parent and child activity levels were compared using paired t-tests. Child activity was compared by sex, age group (8–9 vs. 10–11), acculturation (parent speaks Spanish only vs. parent speaks some English), and seasonality (August–September vs. October–December) using t-tests.

The average minutes per hour spent in each activity level was summarized by time of weekday for children. Periods of the day analyzed were before school (6–7:59 am), during school (8–2:59 pm), afterschool (3–5:59 pm), and evening (6–9:59 pm) with pairwise comparisons using t-tests.

### Qualitative data

#### Interviews

Parents participated in semi-structured interviews lasting 30–90 min in their homes. Trained bilingual CHWs conducted the interviews in the participants’ preferred language, Spanish or English. An interview guide, developed by two qualitative researchers in collaboration with CHWs, was used to explore parent’s beliefs about PA, their PA behavior, their child’s PA behavior, child’s use of media and rules for media use (e.g. TV, video games), types of activities parents and children do together and the barriers and facilitators of those activities (see Supplementary file 1 for the full interview guide). Questions included, “What activities does your family do together (riding bikes, taking walks/hikes)”, “How often are you active in a week”, “How many hours do your children spend in screen time (including computer, games, TV) on a typical weekday.” Interviews were recorded and transcribed verbatim; Spanish interviews were translated into English.

#### Data analysis

Qualitative data were analyzed with content analysis to obtain straightforward description [21, 22]. Two qualitative researchers (CKP and WBA) reviewed and coded all transcripts independently. Each researcher read each transcript in its entirety to obtain an overall sense of the meaning then completed line by line coding. They discussed and compared codes reaching agreement. They categorized codes and identified themes from the categories. During this process, a codebook was developed to document codes and definitions. NVivo (version 12, QSR International, USA) was used to organize codes into categories and themes.

## Results

### Quantitative results

Twenty-five parents and 27 children had complete accelerometer data (5–7 days > 10 h per day) and were included in the quantitative analysis. For children 60% were female and the mean BMI percentile was 75%, corresponding to normal weight. For parents, 96% were female with a mean BMI of 32.7 kg/m2 (Table 1).

**Table 1:** Demographic information of participants

Demographics	Child ***n*** = 27	Parent ***n*** = 25
**Weight in Kg (mean)**	39.7	83.1
**Age in years (mean)**	9.26	37
**Gender (n)**		
Male	13	1
Female	14	24
**BMI**		
BMI Percentile	74.63	N/A
BMI	N/A	32.73
**Birth Place (n)**		
Mexico		23
USA		2
**Household Income (n)**		
Less than 14,999	N/A	5
15,000 to 34,999	N/A	14
35,000 to 50, 000	N/A	6
**Language Spoken**		
Only Spanish	N/A	11
Spanish better than English	N/A	9
Both Spanish and English equally well	N/A	3
English better than Spanish	N/A	1
Only English	N/A	1

**Fig. 1:**
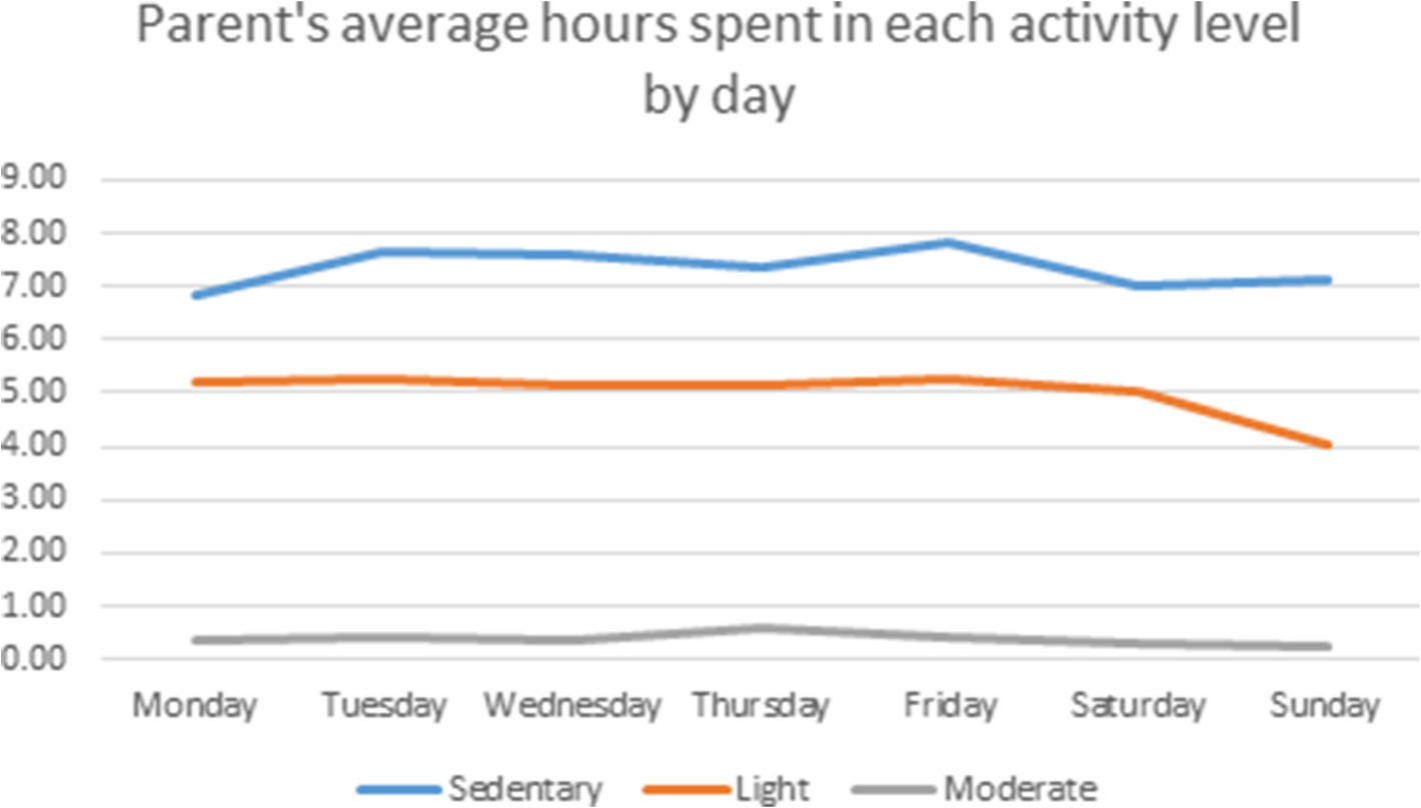
Hours parents (*n* = 25) spent in each activity level per day. Light activity as defined by < 3 METs, moderate between 3 and 5.99 METS [19, 20]

**Fig. 2:**
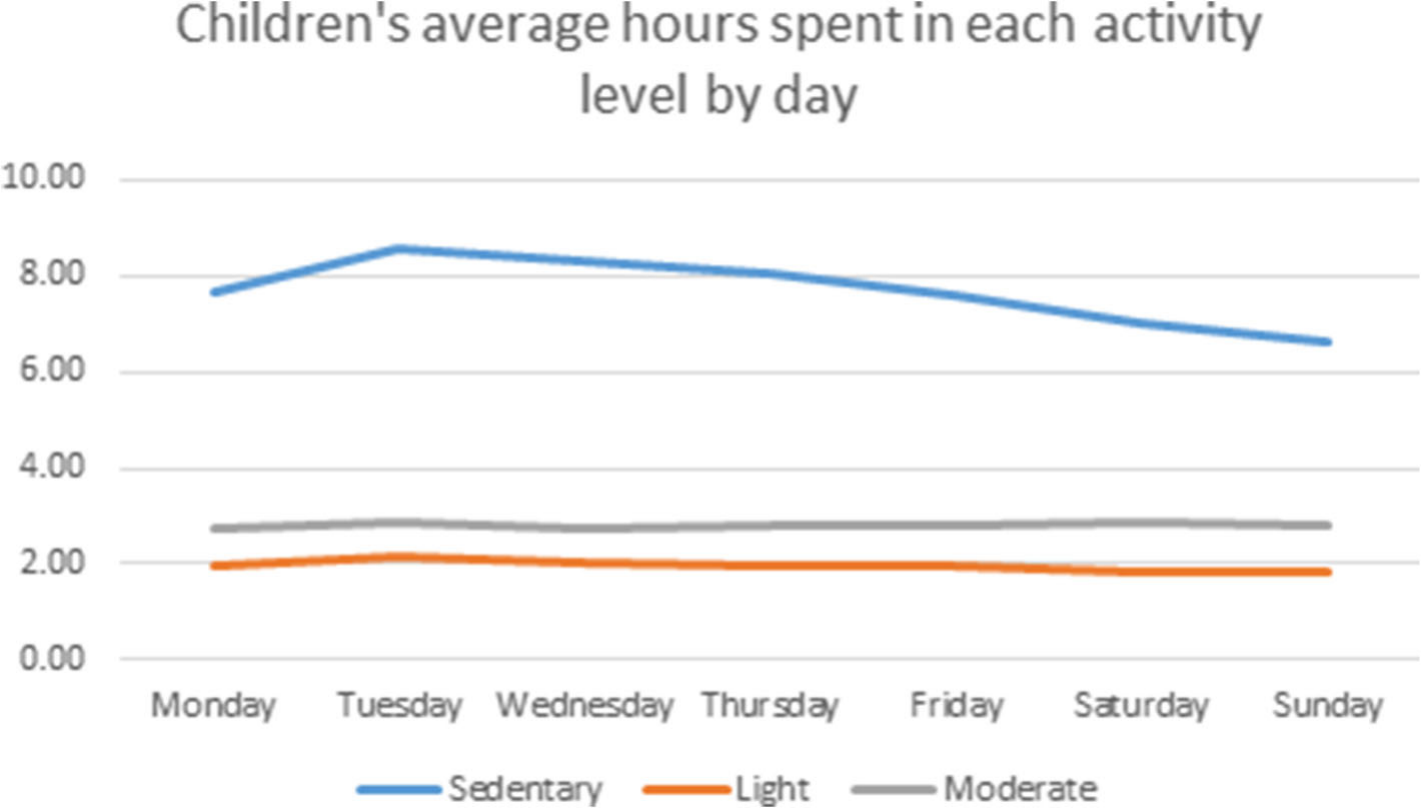
Hours children (*n* = 27) spent in each activity level per day. Light activity as defined by < 3 METs, moderate between 3 and 5.99 METS [19, 20]

All the children (100%) engaged in at least 60 min of moderate-vigorous PA per day and 46% of parents engaged in 150 min of moderate PA per week. On average, parents and children spent similar number of minutes per day, 444 min (7.38 h) and 462 min (7.70 h, *p* = 0.25), being sedentary. Compared to their parents, children spent more minutes in moderate (168 vs. 24, *p* < .0001) and vigorous (7.8 vs. 0 *p <* .0001) activity per day. Parents spent more minutes in light activity per day (299 vs. 118, *p <* .0001; Figs. 1 and 2).

Children spent more time in moderate level activity after school (13.91 min/hour) compared with before school (7.60 min/hour), during school (10.89 min/hour) or in the evening (11.61 min/hour; Table 2). Children spent the most time in sedentary activity during school time (38.50 min/hours) compared with before school (20.26), after school (33.27) and evening (28.82).

**Table 2:** Accelerometer Data. Average minutes per hour children (*n =* 27). spent in each activity level by time of day (weekdays only). Light activity as defined by < 3 METs, moderate between 3 and 5.99 METS, and vigorous as 6.0–8.99 METs [19, 20]

	Before School (6-8 am)		During School (8 am-3 pm)		After School (3-6 pm)		Evening (6-10 pm)	
	Mean Minutes per hour	SD	Mean Minutes per hour	SD	Mean Minutes per hour	SD	Mean Minutes per hour	SD
**Moderate Activity**	7.60	3.44	10.89	2.77	13.91	3.78	11.61	3.53
**Light Activity**	5.05	2.31	8.82	2.51	17.79	2.17	6.82	1.99
**Sedentary**	20.26	9.68	38.50	5.32	33.27	5.84	28.82	6.63

Boys engaged in 4 more minutes/day of vigorous activity compared to girls (*p* = 0.01). There were no differences in activity levels due to acculturation (defined as language spoken by parent) or age group (ages 8–9 and 10–11). Children wearing accelerometers from October–December engaged in 26 more minutes/day of light activity (*p* = 0.005), compared to August–September.

### Qualitative results

Thirty-one parents completed a semi-structured interview. Four themes emerged from the interviews: physical activities and families, barriers and challenges, perceptions and beliefs, and technology and impact on children’s PA.

#### Physical activity and families

Ninety percent of families reported spending time together as a family engaging in PA. The most common activities were walking and spending time at a park. While in the park, parents reported walking, playing soccer or games such as hide and seek, and children playing on equipment (e.g. slides). A few families reported riding bikes together. The majority of parents reported they were active together as a family on weekends.

Almost all parents reported not being active outside of work and chores (we did not collect type or location of PA, thus we are not able to confirm whether parents MVPA was during work of chores only). A few parents reported their children were involved in school sports or leagues. Seventy-five percent of parents reported their children engaged in informal play outside after school and/or rode bikes. As one commented, “I notice that they play here outside with their friends. Outside they’re playing soccer, they’re playing football or running or using bicycles.”

Almost all parents reported that in summer they spent more time outside as a family and their children engaged in more PA; whereas, in winter family time was spent indoors and their children were engaged in sedentary activities (e.g. board games, puzzles). As one commented,

“Well, thinking that it it’s summer, we try to be more outside of the house, running, playing, bicycles. And in the winter, we almost always do a little bit inside of the house. And we try to like to do games like the videogames that now come with dancing or jumping. Not the kind where you just push a button.”

#### Barriers and challenges

For children, the main barriers to being active were homework and being engaged in sedentary activities (e.g. computer games, TV). Children were not engaged in school or league sports because of cost, transportation and not being interested in participating. For parents, barriers included work, lack of time, and lack of opportunities to be active (e.g. no adult sport leagues, facilities). Additionally, chores, childcare and other family/home responsibilities took precedence over being active. As one commented:

“More time. Because thinking about the work schedule, the school schedule, preparing for the next day. It’s too short when you think that one has to prepare everything all the time for the next day. Even though we take some time to have any activity outside but always, I think that sometimes time is too short”.

#### Perceptions and beliefs

While all parents reported PA as being important of overall health, less than half knew what the recommended level of PA was for children and adults. Parents stated that making time for the family to be active together was a promising strategy for increasing PA. Another strategy suggested was for the family to participate in active video games together. Specific health benefits of PA mentioned by parents were reducing weight gain, strengthening bones and muscle strength, improving child physical/emotional development, decreasing stress and improving mood and improving sleep and learning. One summed up:

[Children being active is important] “first to prevent childhood obesity, second when child exercises, he de-stresses, gets tired, learns and goes to sleep well...they have lots of energy, it is good for them, for the development of their muscles to exercise their bodies”.

#### Technology and impact on children’s physical activity

About half of parents reported their children spent 4–6 h per day in screen time including watching TV, using a tablet, playing video/computer games with 33% reporting about 2–3 h per day and 19% about one hour per day. Thirty-three percent of parents reported their children spent more time with screens on the weekends and 9% reported children had less screen time on the weekends because the family engaged in active and sedentary activities together on weekends. Parents expressed concern regarding the impact of screen time on their children; these were children being sedentary, eating more, gaining weight, and harming vision/eyes. As one described:

Television eats their imagination, they stop playing, they stop doing anything to be in front of TV, they stop reading a good book to watch television, or ride the bike to watch television.

Some parents had rules regarding screen time where homework and chores needed to be completed before screen time or limiting number of hours of screen time per day. Many did not have specific rules regarding screen time. A few parents reported that enforcing screen time caused conflict. As one parent succinctly says, “well the kids get mad when I want them to turn off the television.” And another describes how conflict starts with their children, “the kids’ tantrums, the anger, because, ‘why? ‘or ‘give me more time’ or ‘let me watch longer’.” A majority reported watching TV together as a family on the evening and/or weekends. Half of the parents reported their child had a TV in the bedroom.

### Merged quantitative and qualitative results

The merged qualitative and quantitative results are delineated in Table 3. Accelerometry data demonstrated parents and children spent most of each day (weekends and weekdays) in sedentary time, despite parents’ beliefs regarding the potential detrimental effects of sedentary time. Barriers and challenges to meeting PA guidelines, such as lack of opportunities for their entire family, household duties, children’s homework, children’s screen time, and struggles to manage a work-life balance, might explain why parents and children spent much of the day sedentary. Children spent the most time per hour engaged in moderate PA during afterschool hours and children were engaged in informal activity outside during that time.

**Table 3:** Summary of Qualitative and Quantitative Findings

Summary of Activity Patterns	Summary of Qualitative Findings	Interpretation
PA and families: Parents		
• Parents were mostly sedentary • No difference between weekday / weekends activity levels	• Reported being mostly inactive outside of work and household duties • Desired doing PA together as a family by visiting parks on weekends Barriers & Challenges • Lack of time • Work and family responsibilities • Lack of opportunities Perceptions & Beliefs • Identified PA as important for health and wellbeing	• Identified PA as important, yet reported significant barriers to being active on weekdays. • Wanted to be active on the weekends with their families, but minimal activity differences reported between weekends and weekdays.
PA and families: Children		
• Children were mostly sedentary. • Spent most time /hour in moderate activity during afterschool. Hours; 3 – 6 pm • Spent most time /hour in sedentary time during school hours. • No significant differences in PA levels between weekdays and weekends.	• Active with parents when biking, sports, walking and swimming. • Active in informal play during afterschool hours. Barriers and challenges • Lack of other children to be active with. • Health issues such as asthma. • Busy schedules. • Costs of organized team sports and transportation. Perceptions & Beliefs • Parents identified physical activity as important to their children’s health. Impact of technology/ excess screen-time: • Minimal parental limit-setting. • Screen-time as reward • Television in bedrooms • Meals with television	• Parents acknowledged barriers to their children being active despite their support of their children’s PA engagement. • Children engaged in informal play rather than organized team sports afterschool. • Children were on screens an average of 4–6 h per day.

## Discussion

Accelerometry data demonstrated Latino parents and their children spent the majority of the day in sedentary behavior, although 100% of children and 46% adults met PA guidelines. Children spent the most time per hour in moderate level activity during the afterschool hours (3:00 pm – 6:00 pm) compared with other times throughout day. While parents believed in the importance of engaging in regular activity for the health of their families, parents reported barriers to maintaining active lifestyles.

In this study all boys and girls, met the recommended level of daily PA. This is in contrast to other studies which have shown that a majority of rural [7, 8] and Latino [4, 7] children do not meet the national guidelines. This difference might be in part due to informal outdoor play. Both boys and girls had more minutes per hour in moderate PA during after school hours (3 pm–6 pm). Parents reported their children engaged in more informal play outside duirng afterschool hours, playing soccer, football, bicycling and running. Other studies have found greater levels of PA when children are engaged in informal activity outside, including active transport to and from school [23–25].

Children and parents spent most of the day in sedentary behavior. Other studies with Latinos have found an association between Latino children’s and parent’s sedentary behaviors [15, 26]. A study with rural Latinos found that most of family-time activities were sedentary [27]. Sedentary behavior, apart from total PA, is associated with increased cardiovascular morbidity and mortality in adults [28]. In children less is known about the negative effects of sedentary behaviors, however screen time has been associated with adiposity [29]. Organizations have provided sedentary time recommendations for children over five, such as age specific limitations to screen time, but do not give a specific recommendation on amount sedentary behavior per day for health [30, 31].

Parents reported that making time to be active as a family was a promising strategy for increasing PA. In another study, Latino parents suggested “family time” was optimal for engaging in PA [32]. Another strategy parents in our study suggested was participating in active video games as a family. Active video games have the potential to generate energy expenditure comparable to mild to moderate PA intensity for children [32]. However, whether children would engage in sufficient intensity and frequency of active video games to gain the health benefits has not been examined [32]. While active video games may play a role in increasing PA, they come with a significant cost, which may make them difficult to obtain for many families. Supporting Latino families in creating active family time could increase PA in both children and parents.

Despite high levels of children’s PA, parents described barriers both for parents and children. For parents, PA barriers centered on work-related demands, schedules, and lack of available PA opportunities. The high cost and lack of transportation was a barrier to their children engaging in scholastic or league sports. Other studies have identified financial issues (e.g. cost of activity-related equipment, gym memberships, sport league fees), school and work-related activities, seasonal work and transportation issues [33, 34] as barriers for Latino families. This current study as well as other research speaks to a need for low-cost and easily accessible PA opportunities.

Parents reported that screen-time limited opportunities for active play and was a large part of the child’s day (4–6 h). This is consistent with another study which found that Latino children often engage in excessive screen-time more than active play [35] and another that found a strong correlation between Latino children’s inactivity and the amount of time spent watching TV [26]. Additionally, half the parents in our study reported their children had a TV in the bedroom and only 10% reported having rules limiting screen time. This is consistent with research among Latino populations which has shown children tend to have higher rates of TVs located in their bedrooms (74%) than non-Latino children (22%) [26]. Additionally, other studies have shown that a majority of Latino parents do not have rules or limits around screen time [26, 35]. This suggests a need for more resources and guidance on how to limit screen-time. Additional research might explore the difficulties of setting screen-time limits in Latino families.

### Limitations

The study results should be considered in light of the following limitations. The study was conducted with self-identified Latinos living in rural agricultural towns and may not be translatable to other populations due to socioeconomic factors, lifestyles, and cultural considerations. Accelerometer data was collected from August to December and thus did not capture seasonal differences across four seasons. We used primary language spoken by parent as a proxy for acculturation and this measure does not take into account the complexities of acculturation [36] and likely influenced the assessment of the relationship between acculturation and PA. However, this study combined objectively measured physical activity with parent interviews allowing for greater depth of understanding parent influences on PA patterns.

## Conclusion & implications for practice

This mixed methods study provided insight into rural Latino children and parents PA patterns and parent’s influences on their activity can inform interventions aimed at enhancing PA in these families. Participating in PA as a family was an importance value and supporting families in being active together could be an approach to increasing PA in rural Latino families. Addressing barriers (e.g. lack accessible opportunities for PA) and reducing sedentary time could increase PA of rural Latino families.

## Supplementary Information

**Additional file 1.**

## Data Availability

The datasets generated and/or analyzed during the current study are not publicly available but are available from the corresponding author on reasonable request.

## Abbreviations

PA: Physical activity
MVPA: Moderate-vigorous physical activity
CHW: Community health workers
BMI: Body Mass Index
