# Supplementary material for: Rural Latino parent and child physical activity patterns: family environment matters
Source: BMC Public Health. 2021 Nov 8;21:2043. doi: 10.1186/s12889-021-12085-w (PMC8577017; doi:10.1186/s12889-021-12085-w)
Supplement: Supplementary file 1 — Additional file 1. [file 12889_2021_12085_MOESM1_ESM.docx]

**Interview Guide**

**Physical Activity Related Questions**

1. **BRIEF INTRODUCTION**

*“First of all, you can choose a pseudonym (or nick name) and we can use that during the interview.*

*Also, I wanted to remind you that anything we say here today is confidential. We will not be asking any personal questions, only questions related to nutrition and physical activity in your family. None of your responses will be reported outside of this study in a way that could identify you. Only a summary of the all the interviews will be used.*

*Please feel free to share your ideas and opinions with us and remember that there is no right or wrong answer. We just want to learn from you.*

*We would like to record the discussion today to make sure we don't miss any of your comments. We take notes but often they are not as complete as when we record the discussion. If you want to make a comment that you don’t want recorded, just tell us that and we’ll turn off the recorder and re-start it when you finish making your comment. Is that OK?”* **[START THE DIGITAL RECORDER]**

1. **BACKGROUND**

*“Obesity is a serious problem in the U.S. especially among children and adolescents. We want to learn about what is currently happening in terms of nutrition and physical activity among families. We also want to learn about resources that can facilitate healthy eating and help families stay physically active. Our goal is to use this information to develop activities that can help prevent obesity in young people, in our community.”*

1. **PROCESS**

*“I will begin asking some general questions about obesity, nutrition, and physical activity. Then, I will ask you about your family’s nutrition and the type of physical activity that your family takes part in. Finally, I will ask you about any suggestions that you might have on types of family-based activities that could be offered in your community. Okay, let’s start!”*

1. **PHYSICAL ACTVITY RELATED QUESTIONS**
2. What activities does your family do together (for example, riding bikes, taking walks/hikes)?

**[PROBES**:

- Which are your favorite activities?
- Where do you usually go with your family for these activities?
- Who is included in family activities (all kids? parents? Grandparents?)
- Who decides what activities the family will do together?
- Do some family members do some activities together and some do others? (For example, mother and daughter walk together, father and son play ball?) If yes, please specify**]**

1. Does your family play games, such as dominoes together? Can you tell me more about it? How often does your family play together?
2. How often does your family do activities together?

**[PROBES**:

- What designated times or days do you have for family activities?
- Who decides this?**]**

1. Can you tell me about some activities that you only do in the summer or only do in the winter?

**[PROBES**:

- What are these activities?
- When do you do them?
- Are there any other activities?**]**

1. If you could change something about the way you spend time in family activities, what would that be?

**[PROBE**:

- What would help you achieve this? For example, attitudes in family, money, time, places to go, etc…**]**

1. **PHYSICAL ACTIVITY BELIEFS/BEHAVIORS**
2. How much exercise/physical activity does a child need?

**[PROBES**:

- Why is it important for children to be active/exercise?
- Do you think children need to be active everyday? Please explain why you think this.**]**

1. How much physical activity does an adult need?

**[PROBES**:

- Do adults need to be active everyday?
- Why is it important for adults to be active?**]**

1. What kind of sport clubs/leagues/teams do your children participate in? If they don’t participate, why not?

**[PROBES**:

- What types of activities do your children do after school? On weekends? Please tell me more.**]**

1. What kind of organized sport leagues/teams do you participate in? If you don’t participate, why not?
2. How often are you active in a week?

**[PROBE**:

- Would you categorize the exercise as mild (heart rate doesn’t increase) moderate or vigorous (heart rate increases, can perspire)?**]**

1. Could you tell me how you could become more active?

1. Is there anything that prevents you from becoming more active?
2. **MEDIA USE QUESTIONS**
3. How many TVs are in the house?
4. How many computers, laptops, and tablets are in the house?
5. Video games? (such as Nintendo, Xbox)?
6. How many hours do your children spend in screen time (including computer, games, and TV) on a typical weekday?

**[PROBES**:

- Do you think all screen time is the same?
- How is screen time similar or different on weekends?**]**

1. What rules does your family have regarding TV, video games and screen time?

**[PROBES**:

- What information did you use to make the rules?
- How do you enforce the rules?
- What kind of conflict (if any) happens when you enforce the rules?
- Should different children have different rules regarding screen time?
- Can you tell me if you think it’s important to limit screen time, and your reasons for this?**]**

1. How often does your family watch TV together as a family activity?

**[PROBES**:

- Can you describe how often you or your children eat while watching TV, either by themselves or as part of a family group?**]**

1. Who in your house has a TV and/or computer in the bedroom?

**[PROBES**:

- Why are there TVs and/or computers in the bedrooms? Why not?
- If children have TVs and/or computers in their rooms how do you feel about that?
- How do you regulate how much time they spend watching TV and/or using the computer in their rooms?**]**

1. **Check for other ideas and suggestions overall**

*“Do you have additional suggestions for us?”*

1. **CLOSING STATEMENT**

*“That completes the interview. Do you have any other questions, comments, or final thoughts you would like to share?*

*Thank you again for your participation.*

*If you think of other questions, or have any concerns, you can call our office at* 1-866-809-6846*. Before we end the session, I have a few administrative/business details to share with you.*

*Thank you again for this information.”*
